# Assessing knowledge of human papillomavirus and collecting data on sexual behavior: computer assisted telephone versus face to face interviews

**DOI:** 10.1186/1471-2458-9-429

**Published:** 2009-11-23

**Authors:** Anthony Smith, Anthony Lyons, Marian Pitts, Samantha Croy, Richard Ryall, Suzanne Garland, Mee Lian Wong, Eng Hseon Tay

**Affiliations:** 1 Australian Research Centre in Sex, Health and Society, La Trobe University, Melbourne, 3000, Australia; 2 Microbiology and Infectious Diseases Department, Royal Women's Hospital, Melbourne, 3053, Australia; 3 Department of Obstetrics and Gynaecology, University of Melbourne, Melbourne, 3010, Australia; 4 Department of Community, Occupational and Family Medicine, National University of Singapore, 119260, Singapore; 5 Department of Gynaecological Subspecialties and Gynaecological Oncology Unit, KK Women's and Children's Hospital, 229899, Singapore

## Abstract

**Background:**

Education campaigns seeking to raise awareness of human papillomavirus (HPV) and promoting HPV vaccination depend on accurate surveys of public awareness and knowledge of HPV and related sexual behavior. However, the most recent population-based studies have relied largely on computer-assisted telephone interviews (CATI) as opposed to face to face interviews (FTFI). It is currently unknown how these survey modes differ, and in particular whether they attract similar demographics and therefore lead to similar overall findings.

**Methods:**

A comprehensive survey of HPV awareness and knowledge, including sexual behavior, was conducted among 3,045 Singaporean men and women, half of whom participated via CATI, the other half via FTFI.

**Results:**

Overall levels of awareness and knowledge of HPV differed between CATI and FTFI, attributable in part to demographic variations between these survey modes. Although disclosure of sexual behavior was greater when using CATI, few differences between survey modes were found in the actual information disclosed.

**Conclusion:**

Although CATI is a cheaper, faster alternative to FTFI and people appear more willing to provide information about sexual behavior when surveyed using CATI, thorough assessments of HPV awareness and knowledge depend on multiple survey modes.

## Background

The human papillomavirus (HPV) is the most common sexually transmitted infection, and the aetiological agent of cervical, penile, and other anogenital cancers, yet public awareness and knowledge of the virus remains poor[1]. With concerns about further spread of HPV, education campaigns, such as *Healthy People 2010 *in the U.S[2], are trying to change sexual behavior and promote the uptake of HPV vaccination.

To ensure these campaigns remain effective, recent population-based surveys conducted in a range of countries worldwide [3-9] have assessed public levels of HPV awareness and knowledge. Results vary considerably. For example, the percentage of women having heard of HPV ranged from 15% in a Canadian study[8] to 51% in an Australian study[7], while those who were aware of HPV as a risk factor for cervical cancer ranged from 0.9% in the U.K[9] to 34% in Iceland[3]. Although differences in knowledge are likely from one country to another, these studies also varied according to survey mode. Some used mail out surveys[3,5] and face to face interviews (FTFI)[9], while the most recent studies used telephone or computer-assisted telephone interviews (CATI)[4,6-8].

With regard to interviews, it is currently unknown whether CATI and FTFI lead to identical assessments of HPV knowledge and awareness. There are at least two reasons for suspecting differences. First, some subgroups may be less likely to participate depending on the survey mode. For example, mothers with young children may prefer to be interviewed face to face rather than over the telephone because this makes it easier to supervise young children. Those who are particularly busy may prefer CATI over FTFI as it takes less time. Meanwhile, there might be fewer landline telephone numbers for some groups, such as younger people relying entirely on mobile phones. In addition, some people might be more likely to screen their calls than others.

Second, the survey mode can affect how honest and accurate people are when disclosing information depending on the survey topic and the types of questions [10-14]. For example, telephone interviews often facilitate greater disclosure of sensitive information than interviews conducted face to face, due to a greater sense of anonymity[13], but do not always result in people answering honestly[10,14,15]. Of particular concern are questions about sexual behavior. Gauging sexual behavior is often necessary to formulate effective HPV education campaigns, however, due to the highly sensitive nature of these questions, it is essential to choose a survey mode that maximises disclosure but also enables high levels of honesty. Of relevance to assessing HPV knowledge, telephone interviews have also been shown to result in greater time pressure for interviewees than those conducted face to face, which can adversely affect their recall of knowledge[10].

To determine the optimum survey mode for assessing HPV knowledge and related sexual behaviors, a study comparing CATI and FTFI is required. This paper reports a population-based study, conducted in Singapore, where both survey modes were used to assess men's and women's HPV awareness and knowledge, and their related sexual behaviors. Because Singapore is a sexually conservative society, respondents may be particularly sensitive to the type of survey mode when answering questions.

## Methods

### Participants

A list of 9,000 households from Singapore's residential directory, which contains 915,090 households based on latest data[11], was generated using specially designed computer software that ensures every household an equal chance of selection. The first 4,500 households from this list were assigned to CATI. Letters introducing the study were then sent to these households before Singaporean interviewers contacted them by telephone up to three days later. The remaining 4,500 households were assigned to FTFI. Again, letters were sent to these households to introduce the study before interviewers visited their home up to three days later. Women were eligible to participate if aged between 18 and 49 years and men were eligible if aged between 18 and 54 years. Age was the only selection criterion and was used because parents in these groups are most likely to have children at an age for which HPV vaccination is recommended. Given these constraints and the likely situation that not all eligible participants would agree to be interviewed, we expected that contacting 9,000 households would produce a sample size of at least 3,000 women and men, which we determined as necessary for complex epidemiological analyses. In all, 2,145 women and 930 men agreed to participate.

### Survey Instrument

The same questionnaire from a 2007 Australian study[7] on women's understanding of HPV was used in this study, with only slight modifications to account for language and cultural differences. Of relevance to this paper were questions relating to two main themes: 1) awareness and knowledge of HPV (see Table 1) and sexual behavior (see Table 2 and Table 3). To determine whether the survey mode influenced participation from particular demographic groups, a range of other questions were asked relating to the respondents' age, ethnicity, language spoken at home, educational attainment, occupation, and whether they had children under age 16 living with them. A pilot study of 20 participants via CATI was carried out to test the questionnaire.

**Table 1:** Percentage of correct responses to HPV questions by women* and men^§ ^in each survey mode

	Women		Men	
Knowledge items	CATI	FTFI	CATI	FTFI
It is important for all women to be screened for HPV	86.6^a^	86.4^a^	87.8	86.6
HPV is a serious health issue	85.1	87.7	82.9	79.1
Infection can occur via sexual contact	81.4^b^	94.5^b^	87.8	89.6
HPV is associated with abnormal Pap tests	76.2^c^	63.8^c^	72.0^m^	52.2^m^
HPV is the virus that causes genital warts	69.1^d^	60.0^d^	58.5^n^	56.7^n^
Infection does not occur via toilet seats	66.5^e^	76.2^e^	73.2	82.1
Infection can occur via genital to genital skin contact	61.9^f^	65.1^f^	68.3°	61.2°
There is no way to tell if you have HPV	41.8^g^	39.1^g^	43.9	43.3
HPV does not always lead to cervical cancer	41.8^h^	28.9^h^	52.4^p^	32.8^p^
Infection can occur via skin to skin (e.g., fingers, feet)	17.5^i^	20.4^i^	23.2	23.9
HPV cannot be cured	17.5^j^	14.0^j^	34.1^q^	17.9^q^
HPV is not the virus that causes blisters	15.5^k^	12.3^k^	17.1	7.5
HPV is not the virus that causes ulcers or sores	11.9^l^	8.9^l^	15.9^r^	1.5^r^
				
Mean knowledge score (out of 13)	6.73	6.57	7.17^s^	6.34^s^

*N = 429. ^§^N = 147. HPV = human papillomavirus. CATI = computer assisted telephone interview. FTFI = face to face interview. Proportions that share the same superscript differ at p < .05 using chi-square tests

**Table 2:** Percentage of women* and men^§ ^in each survey mode who answered the sexual behavior questions

	Women		Men	
Sexual behavior items	CATI	FTFI	CATI	FTFI
	%	%	%	%
Number of sexual partners over life time	91.0^a^	83.3^a^	85.0	80.7
Number of sexual partners over past 12 months	95.2^b^	84.3^b^	90.2^f^	85.6^f^
Number of current regular sexual partners	94.6^c^	76.9^c^	91.1^g^	74.9^g^
Contraception used during first-time sex	95.6^d^	81.6^d^	92.6^h^	83.2^h^
Age at first sex	89.8^e^	70.2^e^	85.6^i^	70.9^i^

*N = 2145. ^§^N = 930. CATI = computer assisted telephone interview. FTFI = face to face interview. Proportions that share the same superscript differ at p < .05 using chi-square tests

**Table 3:** Answers to sexual behavior questions by those who disclosed, according to gender and survey mode

	Women		Men	
Sexual behavior questions	CATI	FTFI	CATI	FTFI
	%	%	%	%
Total number of sexual partners				
0	25.2^a^	16.6^a^	29.5	29.7
1	62.8^b^	71.1^b^	40.0	46.8
2	7.3	6.8	8.2	8.7
3	1.6^c^	3.1^c^	7.7	5.0
4	0.8	0.9	3.6	2.6
5	1.1	0.5	3.8	2.6
>5	1.3	1.1	7.2	4.5
*From respondents reporting to be sexually active:*				
				
Number of sexual partners over past 12 months				
0	6.6	5.4	13.0	9.3
1	92.6	92.8	79.3	85.2
2	0.7	1.3	5.0	3.8
3	0.0	0.5	1.7	0.3
4	0.0	0.0	0.3	0.0
5	0.0	0.0	0.3	0.3
>5	0.0	0.0	0.3	1.0
Number of current regular sexual partners				
0	1.3	1.8	7.2	5.6
1	98.6	97.5	90.9	93.0
2	0.0	0.3	1.5	1.4
3	0.0	0.3	0.0	0.0
Contraception used with first sexual intercourse				
Yes	32.8	33.2	41.3	40.5
				
	M	M	M	M
Age at first sex	23.9	23.6	24.2	24.1

CATI = computer assisted telephone interview. FTFI = face to face interview.

Proportions that share the same superscript differ at p < .05 using chi-square tests

### Procedure

Ethics approval for this project was granted by La Trobe University Ethics Committee (07-48). Households were randomly assigned to either CATI or FTFI. When interviewers called or visited households, they gave a brief explanation of the study and then determined how many members fell within the target population (i.e., aged 18-49 years for women and 18-54 years for men) before randomly selecting a participant by asking for the person who last had a birthday. Those who agreed to participate had the study fully explained to them before they gave their verbal consent to be interviewed. Of all participants contacted, 43.6% of the FTFI group and 42.8% of the CATI group resulted in a completed interview. All interviews were conducted by female interviewers from a Singapore-based community and health research organization and participants were free to end the interview at any time. Due to the possibility of some respondents terminating the interview when asked sexual behavior questions, these were asked at the end. In addition, those surveyed through FTFI answered the sexual behavior questions in a written self-complete questionnaire to enable greater privacy. Both CATI and FTFI interviews were held between 11 am and 10 pm seven days a week from November, 2007 to January, 2008. Participants chose to be interviewed in English, Chinese, or Malay. In total, 1503 (1044 women, 459 men) interviews were conducted using CATI and 1572 (1101 women, 471 men) using FTFI.

### Statistical Analyses

We first examined the demographic profiles for each survey mode. A series of chi-square analyses were conducted to determine any statistically significant survey mode differences with regard to age, ethnicity, language spoken at home, educational attainment, occupation, and whether any respondents had children under age 16 years living at home with them.

We next examined the number and proportion of men and women in each survey mode who had heard of HPV. Where survey mode differences in levels of awareness were found, multinomial logistic regression was used to determine whether particular demographic variables were significantly associated with HPV awareness, particularly given that some variations were found in the demographic profiles between the survey modes.

Of those who had heard of HPV, we examined their level of knowledge, such as HPV symptoms and modes of transmission. Knowledge scores were calculated based on the number of correct answers to 13 questions (see Table 1). T-tests and analyses of variance (ANOVAs) were conducted to determine any significant differences in knowledge scores with respect to gender and survey mode. Where survey mode differences in HPV knowledge scores were found, multiple regression was used to determine whether particular demographic variables were significantly associated with these scores.

We next assessed the proportion of women and men who were willing to answer five sexual behavior questions and used a series of chi-square analyses to determine any significant survey mode differences in their willingness to answer. Of those who answered the questions, chi-square analyses were again used to assess whether women and men gave different responses depending on the survey mode. Differences between groups in all analyses in this study were treated as statistically significant at p < .05.

## Results

### Demographic Profiles

According to Singapore's 2000 census[12,13], of female residents between 20 and 49 years, 12.8% were aged 20-24 years, 34.2% were 25-34 years; 37.7% were 35-44 years, and 15.5% were 45-49 years. Of male residents between 20 and 55 years, 11.4% were aged 20-24 years, 29.0% were 25-34 years; 34.2% were 35-44 years, and 25.3% were 45-54 years. More than three-quarters (76.8%) of residents were Chinese, 13.9% Malay, 7.9% Indian, and 1.4% had some other ethnic background. Mandarin (35.0%) was the most commonly spoken language at home, followed by a non-Mandarin Chinese language (23.8%), English (23.0%), Malay (14.1%), and Tamil (3.2%). Of those aged above 15 years, 20.0% had no education, 13.1% a primary education, 35.8% a secondary education, 5.0% a vocational qualification, 9.0% a pre-university education, 4.8% a professional diploma, and 12.3% a university degree. No census information about occupation was found that was comparable to data presented in our study.

The demographic profile for our sample was broken down by gender and survey mode and presented in Table 4. The mean age was 35.4 years for women and 34.8 years for men. In all, there were more survey mode differences in the demographic profiles for women than for men. Significantly more women in the CATI group were aged 18-24 years than in the FTFI group while the FTFI group had more women aged 25-34 years (χ^2 ^[3, N = 2145] = 10.47, p < .05). Although both survey groups had approximately equal proportions of women with a Malay background, the CATI group had significantly more Chinese women and the FTFI group significantly more Indian women (χ^2 ^[3, N = 2145] = 12.32, p < .01). There were no significant survey mode differences with regard to language spoken at home. Significantly more women in the FTFI group had lower levels of education compared to those in the CATI group (χ^2 ^[5, N = 2145] = 45.83, p < .001). Meanwhile, the CATI group had significantly more students and white collar workers whereas the FTFI group had more women who reported their occupation as home duties (χ^2 ^[8, N = 2145] = 49.76, p < .001). The FTFI group also had significantly more women with children younger than 16 years compared to the CATI group (χ^2 ^[2, N = 2121] = 7.61, p < .01).

**Table 4:** Percentage of women* and men^§ ^in each demographic according to survey mode

	Women		Men	
	CATI	FTFI	CATI	FTFI
Age group				
18-24	17.4^a^	13.7^a^	22.4	25.5
25-34	23.9^b^	28.2^b^	22.0	26.3
35-44	41.6	39.0	29.0	27.0
>44	17.1	19.1	26.6	21.2
Ethnicity				
Chinese	70.6^c^	66.3^c^	70.2	65.8
Malay	15.8	15.5	15.5	18.3
Indian	9.8^d^	14.7^d^	11.3	13.2
Other	3.8	3.5	3.0	2.8
Language				
English	31.8	29.6	30.5	37.6
Mandarin	41.8	41.7	34.9	30.4
Malay	11.0	12.1	11.3	12.7
Tamil	4.7	7.4	5.4	6.6
Other Chinese	7.9	6.0	14.4	10.0
Other	2.9	3.3	3.5	2.8
Education				
Primary	13.6^e^	22.3^e^	11.3	13.0
Secondary	31.9^f^	35.9^f^	24.8^m^	31.2^m^
Vocational qualification	3.4	3.3	8.3	7.0
Pre-university	13.9	11.0	18.1	15.5
Professional diploma	17.9^g^	13.3^g^	17.9	22.7
University degree	22.7^h^	17.3^h^	27.9^n^	17.6^n^
Occupation				
White collar	51.0^i^	41.2^i^	50.5	46.1
Blue collar	8.2	9.0	24.8	27.6
Self-employed	1.1	1.9	3.5	5.1
Student	11.4^j^	7.1^j^	14.4	13.8
Home duties	26.7^k^	38.8^k^	0.4	0.0
National service	0.0	0.1	3.7	5.5
Other	1.6	1.9	2.6	1.9
Children <16 years old	61.9^l^	67.6^l^	49.6	46.8

*N = 2145. ^§^N = 930. CATI = computer assisted telephone interview. FTFI = face to face interview. Proportions that share the same superscript differ at p < .05 using chi-square tests

For men, educational attainment was the only demographic variable found to significantly vary between the surveys. In particular, more lower educated men were in the FTFI group and more higher educated men were in the CATI group (χ^2 ^[5, N = 930] = 18.28, p < .01).

### Awareness of human papillomavirus

Respondents were asked whether they had heard of HPV, to which they answered "yes', "no", or "don't know". Significantly more women in the FTFI group (21.3%) reported an awareness of HPV than in the CATI group (18.6%) (χ^2 ^[2, N = 2145] = 9.01, p < .05). Although more men in the CATI group (17.9%) reported an awareness of HPV than in the FTFI group (14.2%), this difference was not significant.

Because the demographic composition varied between the survey modes, we used multinomial logistic regression to determine the demographic variables most associated with differences in women's HPV awareness. Educational attainment (F [5, 2094] = 8.65, p < .001) and occupation (F [8, 2094] = 2.05, p < .05) were the only two variables significantly associated with HPV awareness. Awareness tended to increase with greater education (primary = 15.6%; secondary = 15.0%; vocational qualification = 14.7%; pre-university = 22.9%; professional diploma = 23.8%; university degree = 32.2%). Meanwhile, women who were self-employed (29.0%), students (23.1%), or working in white collar occupations (23.8%) were more likely to have heard of HPV than those performing home duties full-time (17.3%) or working in blue collar occupations (11.4%). However, when differences in the demographic profiles between the survey modes were held constant, women in the FTFI group still displayed significantly greater HPV awareness (F [1, 2094] = 8.44, p < .01), therefore suggesting additional reasons for these differences in awareness.

### Knowledge of human papillomavirus

Respondents who had heard of HPV were asked 13 questions to assess their overall knowledge, to which they answered "yes", "no", or "don't know". Table 1 displays the correct answers along with the proportion of women and men who gave these answers. An HPV knowledge score was then computed with one point awarded for each correct response. Out of a possible maximum score of 13, men (M = 6.76) scored slightly higher than women (M = 6.65), but this difference was not significant. Despite a number of significant survey mode differences in the proportion of women correctly answering each knowledge question (see Table 1), an ANOVA revealed no significant differences in women's mean knowledge scores. In contrast, men in the CATI group (M = 7.17) scored significantly higher than those in the FTFI group (M = 6.34)(F [1,146] = 5.81, p < .05).

Because the demographic profiles varied between the survey modes, multiple regression was used to determine the demographic variables most associated with men's knowledge scores. Language spoken at home was the only variable significantly associated with knowledge scores (F [5,122] = 3.25, p < .01), with English speakers (M = 7.46) displaying higher scores than Mandarin (M = 6.37), Malay (M = 6.33), Tamil (M = 6.38), and non-Mandarin Chinese (M = 6.30) speakers. However, even when differences in the demographic profiles between the survey modes were held constant, men in the CATI group still displayed better overall knowledge than those in the FTFI group (F [1,122] = 7.02, p <.01), therefore suggesting additional reasons for survey mode differences in men's knowledge scores.

### Willingness to answer sexual behavior questions

Participants were asked five questions about their sexual behavior, including total number of sexual partners, number of sexual partners over the past 12 months, number of current regular sexual partners, whether they used contraception during first sexual intercourse, and their age when they first had sex. We examined whether their willingness to disclose information to these questions varied according to survey mode.

Turning first to the total number of sexual partners, despite answering sexual behavior questions in a self-complete questionnaire, significantly more women in the FTFI group refused to answer than in the CATI group (χ^2 ^[2, N = 2145] = 30.01, p < .001)(see Table 2). However, this was not the case for men, who showed no significant survey mode differences in their willingness to answer this question.

For all remaining sexual behavior questions, both men and women in the FTFI group were more likely to refuse disclosure than those in the CATI group, although this tendency was greater for women than for men (see Table 2). Chi-square analyses indicated these differences between survey modes were statistically significant for both women (number of sexual partners over past 12 months: χ^2 ^[2, N = 2145] = 68.79, p < .001; number of current regular sexual partners: χ^2 ^[2, N = 2145] = 135.88, p < .001; use of contraception during first sexual intercourse: χ^2 ^[2, N = 2145] = 103.23, p < .001; age when first had sex: χ^2 ^[2, N = 2145] = 134.89, p < .001) and men (number of sexual partners over past 12 months: χ^2 ^[2, N = 930] = 10.31, p < .01; number of current regular sexual partners: χ^2 ^[2, N = 930] = 42.62, p < .001; use of contraception during first sexual intercourse: χ^2 ^[2, N = 930] = 20.09, p < .001; age when first had sex: χ^2 ^[2, N = 930] = 30.59, p < .001).

### Information disclosed to sexual behavior questions

Regarding the total number of sexual partners, women in the FTFI group were significantly more likely to report having at least one sexual partner than those in the CATI group, who were more likely to report not having had a sexual partner (χ^2 ^[8, N = 1867] = 27.36, p < .001)(see Table 3). No such differences were observed for men. Although there were slight variations in responses to the remaining questions between the FTFI and CATI groups, none of these were significant for either women or men.

## Discussion

Consistent with population-based studies conducted in other countries[1], Singaporean men and women displayed overall low levels of HPV awareness and knowledge. However, survey mode differences emerged with regard to women's HPV awareness, both men's and women's knowledge of specific HPV knowledge items, and men's overall HPV knowledge scores. Some of these differences were at least partly explained by variations in the demographic profiles between the survey modes, particularly with regard to education, occupation, and language spoken at home.

However, these differences alone were not sufficient for explaining survey mode differences in awareness and knowledge, particularly evident by the fact that more women in the FTFI group were aware of HPV than in the CATI group despite the FTFI group having fewer highly educated women. Although additional demographic variables not included in our survey may have played a role, it is also possible that some women in the FTFI group falsely reported an awareness of HPV as this survey mode is particularly susceptible to socially-desirable responding[12]. Likewise for men, demographic variations could not fully explain survey mode differences in HPV knowledge scores. Again, demographic variables not included in our study may have been contributing factors, however, it is also possible that a greater sense of anonymity in CATI resulted in more men attempting to guess their answers.

These possibilities and the fact that the survey modes attracted somewhat different demographic profiles despite random sampling suggests that the practical benefits of CATI compared to FTFI, such as being cheaper and faster to administer, need to be weighed against the possibility that data may not be as representative of the population as it could be when combining both survey modes. On this note, differences in the demographic profiles between the survey modes were much greater for women than for men. The FTFI group, for example, had a larger number of women who performed home duties as their only occupation, perhaps because being surveyed face to face makes it easier to supervise young children, whereas full-time workers who have less flexibility may prefer the speed and convenience of CATI.

Also, given that neither survey mode produced a demographic profile that exactly matched the Singaporean 2000 census data, highly accurate assessments of HPV awareness and knowledge may not be possible using either mode without employing sample matching techniques or weighting the data. In particular, both survey modes were overrepresented by women and men with higher education levels, so future studies will need to at least find ways of increasing the representation of the poorer educated.

With regard to sexual behavior, lowered willingness to answer the sexual behavior questions among those surveyed via FTFI compared to CATI was consistent with past research[14]. Even having respondents answering sensitive questions in a self-complete questionnaire still resulted in lower disclosure in FTFI than CATI, perhaps suggesting that any face to face component in a survey reduces a respondent's sense of anonymity. The fact that respondents appear more open to answering sexual behavior questions through CATI than FTFI may also lead to greater accuracy of data. Although we had the benefit of a large sample and therefore recorded few survey mode differences in the types of responses given by women and men, studies using smaller samples may find FTFI leads to such a low response rate when asking sensitive questions that it substantially weakens the reliability of their findings.

Of course, the results of this study are limited by the HPV and sexual behavior questions we chose to include. Had we examined additional HPV knowledge and sexual behavior questions, overall survey mode differences may have been different. The inclusion of additional demographic variables, such as socioeconomic status, family structure, or residential location, might have also enabled us to better account for survey mode differences in HPV awareness and knowledge.

It is also worth noting that less than half of those contacted were actually interviewed. Given the over-representation of highly educated women and men in our sample, it is possible that those with lower education were less willing to participate. Since HPV awareness appears to be greater with higher levels of education, it is therefore likely that Singaporean's overall awareness is substantially lower than our findings would indicate.

One further limitation of this study was the fact that more than twice as many women as men were interviewed, therefore giving much greater statistical power to the women's sample. This may at least partly explain why more significant survey mode differences were found between the HPV knowledge items for women than for men. Although men in our study still comprised a relatively large sample, being substantially smaller makes it more vulnerable to sampling bias. For example, there were more younger men than younger women, evidenced by a greater proportion of men in the 18-25 age group and an overall lower mean age despite including men up to the age of 54 years. Despite this, much of the research on HPV awareness and knowledge shows no age differences[1]. Meanwhile, for many other demographic variables, differences in the proportions of men compared to the census data appeared no greater than those for women, which suggests that any tendency for the men's sample to be more vulnerable to sampling biases than the women's sample was limited.

## Conclusion

Conducting research using CATI is less expensive and faster than FTFI, so a strong case can be made for favouring this survey mode, especially as it leads to higher disclosure for sexual behavior. However, although CATI has become the preferred method for assessing public HPV awareness and knowledge, the present study suggests the overall accuracy of data on this topic is potentially compromised by using a single survey mode. Campaigns aiming to reduce HPV transmission, and therefore cervical and other cancers, by educating the public about the virus require comprehensive and accurate assessments of knowledge items most lacking in the community. For this reason, future research in this field is likely to benefit from using mixed-mode surveys while ensuring all relevant demographics are fully represented. Future research should also explore the effect of additional survey modes on assessing HPV awareness and knowledge, including sexual behavior, such as mail out questionnaires, web-based surveys, computer-assisted self interviewing[15,16], and automated telephonic data collection[13] to further determine the optimum method.

## Competing interests

GlaxoSmithKline (GSK) awarded funds to Suzanne Garland to support this research in the interests of informing their commercial decisions regarding HPV vaccination. GSK, however, had no involvement with the design or data analysis of this study. All other authors declare that they have no competing interests.

## Authors' contributions

AS, MP, SC, SG, MLW, and EHT designed the study. AS, MP, and SC supervised the project. AS, AL, MP, SC, and RR analysed and interpreted the data. AL wrote the manuscript and all authors contributed to the final draft. All authors approved this manuscript.

## Pre-publication history

The pre-publication history for this paper can be accessed here:

http://www.biomedcentral.com/1471-2458/9/429/prepub
